# Chemical Properties of Vitis Vinifera *Carménère* Pomace Extracts Obtained by Hot Pressurized Liquid Extraction, and Their Inhibitory Effect on Type 2 Diabetes Mellitus Related Enzymes

**DOI:** 10.3390/antiox10030472

**Published:** 2021-03-17

**Authors:** Nils Leander Huamán-Castilla, David Campos, Diego García-Ríos, Javier Parada, Maximiliano Martínez-Cifuentes, María Salomé Mariotti-Celis, José Ricardo Pérez-Correa

**Affiliations:** 1 Escuela de Ingeniería Agroindustrial, Universidad Nacional de Moquegua, Moquegua 18001, Peru; nhuamanc@unam.edu.pe; 2 Instituto de Biotecnología, Universidad Nacional Agraria la Molina, Lima 15026, Peru; dcampos@lamolina.edu.pe (D.C.); 20100455@lamolina.edu.pe (D.G.-R.); 3 Institute of Food Science and Technology, Faculty of Agricultural Sciences, Universidad Austral de Chile, Valdivia 5110566, Chile; javier.parada@uach.cl; 4 Centro Integrativo de Biología y Química Aplicada (CIBQA), Escuela de Tecnología Médica, Facultad de Salud, Universidad Bernardo O’Higgins, Santiago 8370993, Chile; maximiliano.martinez@ubo.cl; 5 Escuela de Nutrición y Dietética, Universidad Finis Terrae, Santiago 7501015, Chile; 6 Chemical and Bioprocess Engineering Department, School of Engineering, Pontificia Universidad Católica de Chile, Santiago 7820436, Chile

**Keywords:** *carménère* pomace, hot pressurized liquid extraction, glycerol, ethanol, α-amylase, α-glucosidase

## Abstract

Grape pomace polyphenols inhibit Type 2 Diabetes Mellitus (T2DM)-related enzymes, reinforcing their sustainable recovery to be used as an alternative to the synthetic drug acarbose. Protic co-solvents (ethanol 15% and glycerol 15%) were evaluated in the hot pressurized liquid extraction (HPLE) of Carménère pomace at 90, 120, and 150 °C in order to obtain extracts rich in monomers and oligomers of procyanidins with high antioxidant capacities and inhibitory effects on α-amylase and α-glucosidase. The higher the HPLE temperature (from 90 °C to 150 °C) the higher the total polyphenol content (~79%, ~83%, and ~143% for water-ethanol, water-glycerol and pure water, respectively) and antioxidant capacity of the extracts (Oxygen Radical Absorbance Capacity, ORAC), increased by ~26%, 27% and 13%, while the half maximal inhibitory concentration (IC_50_) decreased by ~65%, 67%, and 59% for water-ethanol, water-glycerol, and pure water extracts, respectively). Water-glycerol HPLE at 150 and 120 °C recovered the highest amounts of monomers (99, 421, and 112 µg/g dw of phenolic acids, flavanols, and flavonols, respectively) and dimers of procyanidins (65 and 87 µg/g dw of B1 and B2, respectively). At 90 °C, the water-ethanol mixture extracted the highest amounts of procyanidin trimers (13 and 49 µg/g dw of C1 and B2, respectively) and procyanidin tetramers of B2 di-O-gallate (13 µg/g dw). Among the Carménère pomace extracts analyzed in this study, 1000 µg/mL of the water-ethanol extract obtained, at 90 °C, reduced differentially the α-amylase (56%) and α-glucosidase (98%) activities. At the same concentration, acarbose inhibited 56% of α-amylase and 73% of α-glucosidase activities; thus, our grape HPLE extracts can be considered a good inhibitor compared to the synthetic drug.

## 1. Introduction

*Carménère* is recognized as Chile’s emblematic wine due to its particular color, aroma, and astringency [1,2]. This wine generates ~80,000 tons of grape pomace (skin and seed), a solid organic byproduct representing a severe environmental problem [3]. After winemaking, *Carménère* pomace retains around 60% of the original polyphenols in the grape berry [4], which contains high amounts of malvidin (anthocyanin), quercetin (flavonol), and epigallocatechin (flavanol), as well as proanthocyanidins [5,6].

Due to the polyphenols’ ability to form complexes with metal ions and macromolecules such as polysaccharides and proteins [7], they are an attractive option to develop nutraceuticals and functional food ingredients [8]. In particular, proanthocyanidins have been shown to inhibit the key enzymes (α-amylase and α-glucosidase) related to Type 2 Diabetes Mellitus (T2DM), being a natural alternative to the synthetic drug acarbose [9,10,11].

Acarbose has been validated to exert an anti-postprandial hyperglycemia effect. However, it causes undesirable side-effects such as flatulence and diarrhea, with corresponding abdominal pain and a loss of nutrient absorption [12].

The biological effect of polyphenols is related to their degree of polymerization (DP), structural units, and substituted groups; however, there are no concluding remarks regarding these structural features’ effects on the differential inhibition of these enzymes [13].

Proanthocyanidins have a high degree of polymerization (DP) and numerous hydroxyl groups compared with other monomeric polyphenols, explaining their higher binding affinity to the digestive starch enzymes [12].

The proanthocyanidins with a DP higher than eight that are present in ripe fruits showed more potent inhibition of α-amylase and α-glucosidase than the less-polymerized proanthocyanidins present in unripe fruits [14]. The low DP proanthocyanidins obtained from green tea also have high inhibitory activity against α-glucosidase [11]. Similarly, trimers of proanthocyanidins obtained from a Chinese baby berry showed the highest inhibition effect on α-amylase and α-glucosidase [12]. Consequently, an efficient and sustainable process to obtain extracts rich in proanthocyanidins is desirable to commercially produce functional ingredients that effectively inhibit the T2DM-related enzymes.

Hot Pressurized Liquid Extraction (HPLE) is a clean technology that overcomes most of the limitations of atmospheric polyphenol extraction [15,16,17]. Pure water is the most used solvent in HPLE to obtain polyphenols [18,19]. However, high extraction temperatures (≥120 °C) degrade polyphenols, forming toxic compounds and increasing the recovery of sugars [19,20].

In the HPLE of polyphenols, protic co-solvents—such as ethanol and glycerol—have been successfully used to reduce the temperature and improve the selectivity of the extraction [21,22,23]. Besides this, HPLE with water-glycerol mixtures yields higher recoveries of some monomers (flavonols, flavanols, and phenolic acids) than water-ethanol mixtures [21,22]. However, these generally recognized as safe (GRAS) solvents’ impacts on the HPLE extraction of proanthocyanidins has not been evaluated yet.

This study hypothesizes that the HPLE of *Carménère* pomace using protic co-solvents such as ethanol and glycerol allows us to obtain extracts rich in proanthocyanidins, which show an inhibitory effect on T2DM-related enzymes comparable to the synthetic drug acarbose.

Herein, the objective of this research was to evaluate the effect of using pure water, water-ethanol, and water-glycerol mixtures (15%) at high temperatures (90, 120, and 150 °C) on the antioxidant capacity and the content of specific monomers, dimers, trimers, and tetramers of the proanthocyanidins of *Carménère* pomace extracts obtained by HPLE. Additionally, the inhibitory effect of these extracts on α-amylase and α-glucosidase was determined and compared with acarbose.

## 2. Materials and Methods

### 2.1. Grape Pomace

In total, ~5 kg of skin and seed (mixture) from *Carménère* pomace was obtained from the Concha y Toro Vineyard in Chile after the winemaking process, and was immediately stored at −20 °C. Before the experimental tests, the grape pomace was reduced down to a ~2 mm diameter using a cutting mill Oster blender (Sunbeam Products, Inc., Boca Raton, FL, USA).

### 2.2. Chemicals and Analytical/Experimental Reagents

The standards of gallic acid (98%), catechin (≥97%), epicatechin gallate (≥97%), kaempferol (≥97%), quercetin (≥95%), epicatechin (≥98%), rutin (≥94%), procyanidin B1 (≥90%) and procyanidin B2 (≥90%) were purchased from Sigma Aldrich Chemical Co. (St. Louis, MI, USA). The solvents glycerol (≥98%) and ethanol (≥99%) were purchased from Winkler Ltd. (Santiago, Chile). The reagents Folin-Ciocalteu, sodium hydroxide, chlorhydric acid, DPPH (2,2-Diphenyl-1-picrylhydrazyl), and Trolox were purchased from Sigma Aldrich Chemical Co. (St. Louis, MI, USA).

### 2.3. Hot Pressurized Liquid Extraction (HPLE) of Carménère Pomace

Approximately 5 g dw *Carménère* pomace was mixed with 110 g quartz sand. The mixture was then placed in an HPLE device (ASE 150, Dionex, Sunnyvale, CA, USA), which was used to obtain the polyphenol extracts from the *Carménère* pomace using pure water, water-glycerol (15%), and water-ethanol (15%) mixtures at 90, 120, and 150 °C. Both the temperature and co-solvent levels were defined based on previous research [21,22]. The extraction conditions were 10 MPa, one extraction cycle, 150% washing volume, 250 s nitrogen purge time, and 5 min static extraction time to obtain a matrix/extractant ratio of 1:10. The collected extracts were transferred to amber vials and stored at −20 °C until the chemical analysis.

### 2.4. Total Polyphenol Content (TPC)

The TPC determination was carried out according to the method proposed by Singleton and Rossi [24]. In total, 3.75 mL distilled water, 0.5 mL sample, and 0.25 mL Folin-Ciocalteau reagent were mixed with 0.5 mL Na_2_CO_3_; the reaction was kept in the dark for one hour. Then, the absorbance was measured at 765 nm. The TPC was expressed as the gallic acid equivalent (GAE) per gram of dry weight, considering a standard curve of gallic acid (10 mg/L−90 mg/L; r^2^: 0.9987).

### 2.5. Antioxidant Capacity by DPPH

The antioxidant activity was determined using DPPH as a reactive radical according to the methodology proposed by Brand-Williams, Cuvelier, and Berset [25]. The IC_50_ of the sample was measured as the polyphenol concentration necessary to inhibit 50% of the DPPH radical activity.

### 2.6. Antioxidant Capacity by Oxygen Radical Absorbance Capacity (ORAC)

The extracts’ antioxidant activity was determined according to the methodology proposed by Chirinos et al. [26]. The ORAC analyses were performed in a 96-well microplate fluorometer (Ascent F.L. Fluoroscan, Labsystem, Finland). 2,2’-Azobis(2-amidinopropane) dihydrochloride (AAPH) (153 mM) was used as a peroxyl radical generator. Trolox (0.01M) was used as the standard, and fluorescein (55 mM) was used as a fluorescent probe. In total, 25 µL of phosphate buffer (75 mM) at pH 7.4 was used as the blank. After this, the Trolox standard or the diluted sample in phosphate buffered saline (PBS) buffer at pH 7.4 were mixed with 250 µL of fluorescein and incubated for 10 min at 37 °C. An automatic injection of 25 µL AAPH solution (153 mM) was added to all of the microplates. The fluorescence was measured every minute for 50 min. The final ORAC values were calculated using the area under the curves and were expressed as µmol of Trolox equivalents per gram of dry weight (µmol TE/g dw).

### 2.7. Quantification of the Target Polyphenols

The quantification of polyphenols was performed using the method described by Campos et al. [27], with some modifications. The chromatographic separation was performed on an Ultimate 3000 Ultra high-performance liquid chromatography (UHPLC) system (Thermo Scientific, Germany), connected to a Quantum Access Max triple quadrupole mass spectrometer equipped with a heated electrospray ionization (HESI-II) probe (Thermo Scientific, USA). The HPLE extracts were cleaned up on Sep-Pak C18 cartridges (Waters) before their injection. The samples were injected into an Acquity BEH C18 column (2.1 × 100 mm, 1.7 µm, Waters, Ireland). The mobile phase was composed of water (A) and acetonitrile (B), containing 0.1% formic acid. The total running time was 42 min with the following solvent gradient: 2% B for the first 2.5 min, 7% B for the next 2.5 min, 12% B for the next 12.5 min, 26% B for the next 6 min, 55% B for the next 6 min, 95% B for the next 1 min, kept at this condition for 3.5 min, and in 1 min returned to 2% B. The column was re-equilibrated for 7 min before the next injection. The injection volume was 1 µL. The column was heated at 30 °C, and the flow rate was 0.25 mL/min. The mass spectrometer parameters were the following: spray voltage, −3500 V; sheath gas pressure, 30 (arbitrary units); auxiliary gas pressure, 5 (arbitrary units); ion sweep cone gas pressure, 0.5 (arbitrary units); vaporizer temperature, 378 °C; capillary temperature, 295 °C; collision gas pressure, 1.5 mTorr. The data were acquired using the selected reaction monitoring (SRM) mode. The collision energy was optimized for each compound using the TSQTune software (Thermo Scientific). The phenolic standards (catechin, epicatechin, epicatechin gallate, gallic acid, kaempferol, procyanidin B1, procyanidin B2, quercetin, and rutin) were mixed and diluted to concentrations of 0.01, 0.05, 0.10, 0.25. 0.50, 1.00, 2.50, and 5.00 µg/mL. The calibration curves were constructed by plotting the peak area of the standard against its concentration. The limits of detection (LOD: 0.0003−0.018 µg/mL) and quantification (LOQ: 0.011−0.053 µg/mL) were established for all of the quantified polyphenols. The compounds of which the standards were not available (trimers and tetramers) were quantified as equivalents of a known standard with a common *m/z* fragment.

### 2.8. α-Amylase Activity Inhibition

The ability of each extract to inhibit α-amylase activity was measured using the method described by Pacheco et al. [28]. For the samples’ preparation, each extract obtained was dried with nitrogen gas, resuspended in DMSO (dimethylsulfoxide), and filtered to obtain 10 mg *Carménère* pomace’s stock solution. The assay dilutions were made from this stock: 0.1−1000 µg of *Carménère* pomace/mL phosphate buffer (pH 6.9). In total, 100 μL of each sample dilution and 1% starch solution in 20 mm sodium phosphate buffer (pH 6.9 with 6 mm sodium chloride) were incubated in microtubes at 25 °C for 10 min in a water bath. Then, 100 μL of porcine pancreatic α-amylase (0.5 mg/mL) was added to each tube, and the samples were incubated at 25 °C for another 10 min. After the reaction finished, 200 μL dinitrosalicylic acid reagent was added. After that, the tubes were incubated at 100 °C for 5 min in a water bath. Subsequently, 50 μL of each reaction mixture was transferred to a 96-well microplate and diluted by adding 200 μL water to each well, and the absorbance was measured at 540 nm. The enzymatic activity was determined as follows:

$$\mathrm{Enzymatic}\;\mathrm{activity}\;\left(\%\right)=\frac{\mathrm{Absorbance}\;\mathrm{of}\;\mathrm{extract}\;}{\mathrm{Absorbance}\;\mathrm{of}\;\mathrm{control}\;}\times \;100$$

where the control is the enzyme-substrate reaction in the absence of inhibitors. The effect of the pharmacological inhibitor, acarbose, was also determined following the same protocol previously described.

### 2.9. α-Glucosidase Activity Inhibition

Each extract’s ability to inhibit α-glucosidase activity was measured using the method described by Nampoothiri et al. [28]. The samples were prepared in the same way as for the α-amylase activity assay. Each extract’s inhibitory effect was measured at concentrations from 0.1 to 1000 μg of *Carménère* pomace/mL in 100 mm sodium phosphate buffer (pH 6.9). A volume of 50 μL of the extract solution and 50 μL 5 mm p-nitrophenyl-α-d-glucopyranoside (PNPG) solution (in phosphate buffer) was mixed in a 96-well microplate and incubated at 37 °C for 5 min. Then, phosphate buffer (100 μL) containing 0.1 U/mL of α -glucosidase (from S. cerevisiae) was added to each well. The absorbance at 405 nm was recorded for 15 min using a microplate reader at 37 °C. The commercial inhibitor’s effect on α-glucoside activity was also determined, and the data were processed as in the previous assay.

### 2.10. Statistical Analysis

A factorial experimental design was applied in order to determine the effect of the temperature and solvent during the HPLE of *Carménère* pomace on the antioxidant properties and the recovery of the specific polyphenols of the obtained extracts. Furthermore, the mean and coefficient of variation (CV) results are presented. Analysis of variance (ANOVA) and the least significant difference tests were applied to the response variables (*p* ≤ 0.05). The statistical analyses of the data were carried out using Statgraphics Plus for Windows 4.0 (Statpoint Technologies, Inc., VA, USA).

## 3. Results and Discussion

### 3.1. Effect of the HPLE Conditions on the Antioxidant Properties of Carménère Extracts

#### 3.1.1. Total Polyphenol Content and Antioxidant Capacity

The use of protic co-solvents such as ethanol and glycerol in the HPLE of *Carménère* significantly increased the total polyphenol content (~83%) and antioxidant capacity (DPPH: ~190% and ~227%, respectively, and ORAC: 26%) of the extracts compared to pure water. In subcritical conditions, the polyphenol solubilities in pure water increase. The use of protic co-solvents like glycerol and ethanol reduces the solvent polarity (π*) and favors its ability to form hydrogen bonds (α), improving the solubility and extractability of polyphenols [29] significantly. During HPLE, the operational conditions (temperature and co-solvent addition) alter the solvent’s properties, facilitating the extraction of total and specific polyphenols [21,22].

For all of the extraction solvents evaluated, when the temperature increased from 90 to 150 °C, the total polyphenol content increased by ~79%, ~83%, and ~143% for ethanol, glycerol, and pure water, respectively (Table 1). Similar results were found by Vergara-Salinas et al. [19] and Mariotti−Celis et al. [30] using pure water as the HPLE solvent, enhancing the recoveries by between 42% and 52% when the temperature was increased from 90 °C to 150 °C.

The higher the HPLE temperature, the higher the antioxidant capacity of the extracts (Table 2). When the HPLE temperature increased from 90 °C to 150 °C, the extracts’ antioxidant capacity—as evaluated by ORAC—increased by ~26%, 27%, and 13%, and the IC_50_ values decreased by ~65%, 67%, and 59% for the water-ethanol, water-glycerol, and pure water extracts, respectively (Table 2).

**Figure 1:**
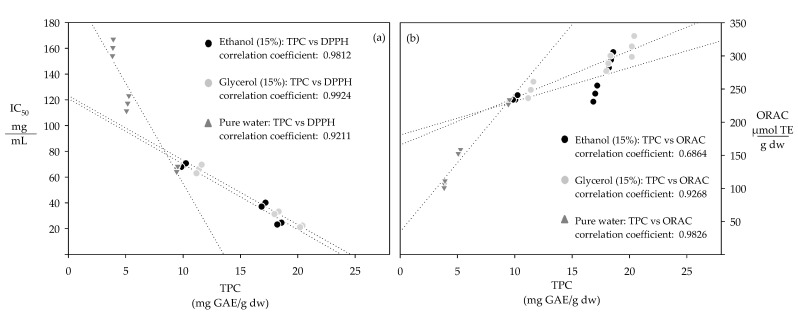
Correlation between the total polyphenol content and the antioxidant capacity represented by, (**a**) IC_50_ and (**b**) ORAC.

The antioxidant capacity of the extracts increased with their total polyphenol content, showing high correlation coefficients between TPC and the corresponding IC_50_ values (0.921–0.992) and ORAC activities (0.686–0.983) (Figure 1).

These complementary assays evaluate the reaction between polyphenols and specific radicals [31]. ORAC measures the polyphenols’ ability to transfer hydrogen atoms to oxygen radicals, while DPPH measures the electron transfer reaction between DPPH radical and polyphenols [32,33]. The ORAC antioxidant activity of the extracts increased proportionally with their total polyphenol content, indicating that the extracts with a higher total polyphenol content presented a higher ability to transfer hydrogen atoms to oxygen radicals [31]. The IC_50_ values of the extracts decreased proportionally with their total polyphenol content; hence, the higher the total polyphenol content, the lower the corresponding IC_50_ value, because a lower amount of extract was needed to inhibit 50% of the DPPH radical [32,33].

#### 3.1.2. Monomers of Flavanols, Flavonols, and Phenolic Acids

**Figure 2:**
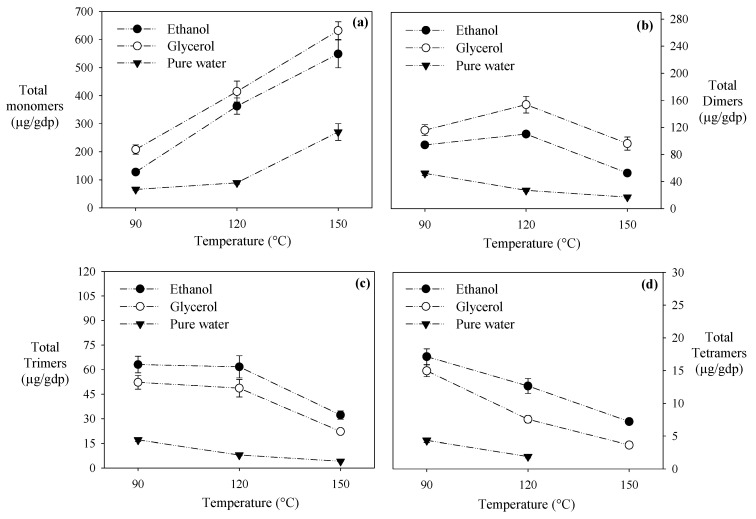
Effect of temperature and solvent extraction on the polyphenols obtained by HPLE. (**a**) Total monomers, (**b**) total dimers, (**c**) total trimers, and (**d**) total tetramers.

Protic co-solvents significantly improved the monomers’ recovery of flavanols, flavonols, and phenolic acids compared to pure water at all the evaluated temperatures. The water-ethanol and water-glycerol mixtures recovered ~2 and ~3 times more total monomers than pure water, respectively (Figure 2). Following the same trend observed for the total polyphenol content and antioxidant capacity in all the evaluated extraction mixtures, the higher the HPLE temperature, the higher the monomers’ recovery (Figure 1a). Rutin is an exception; an increment from 120 °C to 150 °C significantly reduced (>95%) its recovery for all the solvents (Table 3). Similarly, in the water-ethanol (50%) HPLE of apple pomace, rutin’s recovery decreased ~65% when the extraction temperature increased from 100 °C to 135 °C [34]. It has been reported that rutin suffers from thermal degradation above 100 °C [35].

Both water mixtures of glycerol and ethanol recovered similar amounts of gallic acid, although water-glycerol recovered more flavonols (quercetin, kaempferol, and rutin) than water-ethanol (Table 3). Previously, we found that water-glycerol mixtures have a higher ability to form hydrogen bonds with the flavonols´ carbonyl and hydroxyl groups than water-ethanol mixtures. This ability is due to the three hydroxyl groups in glycerol’s chemical structure [21,22]. Moreover, the extracts with the highest flavanols content (catechin ~204 µg/g dw, epicatechin gallate ~54 µg/g dw) were obtained at 150 °C with water-glycerol mixtures (Table 3). These results are also due to the glycerol´s chemical structure’s ability to interact with catechin and epicatechin gallate [22,29,36].

#### 3.1.3. Dimers, Trimers, and Tetramers of Procyanidins

The water-glycerol mixtures recovered more dimers of procyanidin at all of the extraction temperatures than water-ethanol mixtures and pure water (Figure 2). The highest recovery of dimers (153.64 µg/g dw) was achieved with water-glycerol mixtures at 120 °C, while higher temperatures (150 °C) decreased it by ~37% (Table 3). Mauromoustakos et al. [16] reported that water-ethanol mixtures at 140 °C recovered ~0.4 times more dimers of procyanidins than pure water under HPLE conditions. Procyanidin B2 was the most extracted dimer in our study, reaching concentrations of ~56 µg/g dw, ~49 µg/g dw, and ~7 µg/g dw with water-glycerol, water-ethanol, and pure water, respectively (Table 2). Probably, the high number of hydroxyl groups in procyanidin B2 favors the formation of hydrogen bonds with the hydroxyl groups in glycerol.

In previous studies, we found that temperatures higher than 90 °C promote proanthocyanins’ hydrolysis [19,30]. Here, we confirmed these findings, observing that an extraction temperature increases from 90 to 150 °C considerably reduced the content of trimers and tetramers of procyanidins: between 58% and 60% for water-ethanol extracts, between 57% and 76% for water-glycerol extracts, and between 72% and 100% for pure water extracts (Table 3).

Interestingly, when the HPLE was performed at 90 °C, the water-ethanol mixture extracted 15% more trimers and 13% more tetramers than the water-glycerol mixture (Figure 1). This ethanolic extract contained 55.81 µg/g dw of procyanidin B2 gallate, 17.32 µg/g dw of procyanidin C1, and 17.13 µg/g dw of procyanidin di-O-gallate (Table 3). Less polar solvents have been shown to be more effective in recovering trimers and tetramers of procyanidins. These high molecular weight polyphenols have long hydrocarbon chains that prefer ethanol instead of glycerol to form hydrogen bonds [37].

Hence, to efficiently extract the trimers and tetramers of procyanidins using HPLE, moderate temperatures and water-ethanol mixtures are recommended. In turn, to favor the extraction of monomers and dimers, water-glycerol mixtures at high temperatures should be considered.

### 3.2. Inhibition of the Enzymatic Activity

#### 3.2.1. Inhibition of α-Amylase by *Carménère* Pomace Polyphenol Extracts

**Figure 3:**
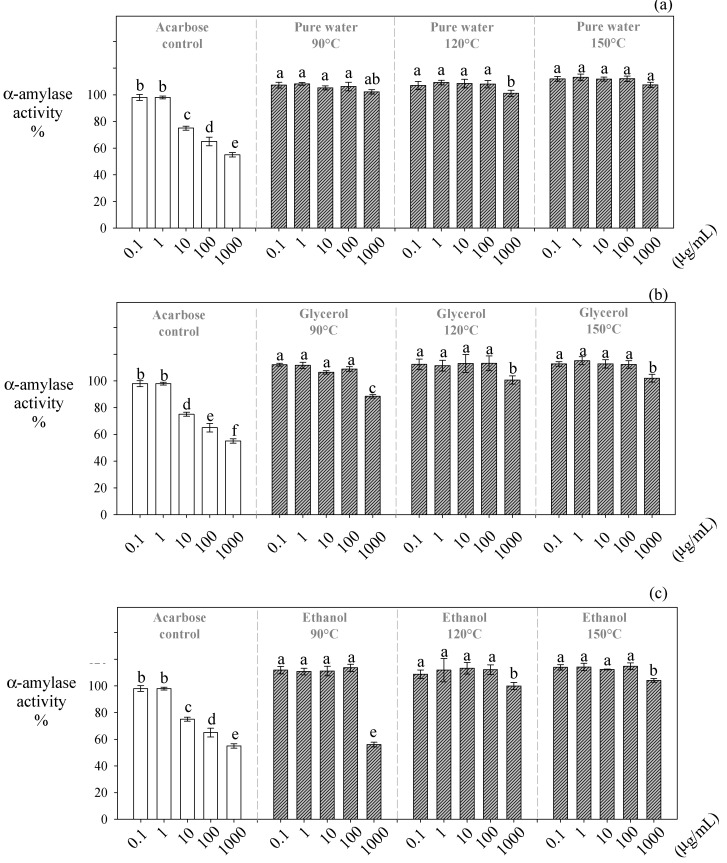
Inhibitory effect of *Carménère* pomace extracts obtained by HPLE at different temperatures using protic solvents on the α-amylase activity. (**a**): pure water, (**b**): glycerol, and (**c**): ethanol. The different low-case letters in the bars show the significant inhibitory differences (*p* < 0.05) between the different HPLE temperatures and extract concentrations for the same HPLE solvent.

In this study, the extracts obtained at higher temperatures presented the lowest inhibitory effect on α-amylase (Figure 3). The inhibitory effect of the water-glycerol and water-ethanol extracts on α-amylase was significantly reduced (~13% and ~46%, respectively) when the temperature of the extraction was increased from 90 °C to 150 °C (Figure 2). High temperatures (>120 °C) promoted a higher recovery of monomers of phenolic acids, flavanols, and flavonols. However, the recovery of procyanidins was significantly decreased (Table 3), disfavoring the extracts’ α-amylase inhibitory effect. Water-ethanol extracts (1000 µg /mL) obtained at 90 °C were the most effective at inhibiting α-amylase (56%), presenting an effect similar to acarbose (Figure 3a). Interestingly, these extracts contained the highest amounts of trimers (63.13 µg/g dw) and tetramers (17.13 µg/g dw) of procyanidins (Table 3).

Procyanidins with a DP higher than two have shown a higher inhibition effect on α-amylase than their monomers [38]. Probably, the interaction between procyanidins and α-amylase promotes the formation of procyanidin/α-amylase complexes that reduce its activity [11,39]. A recent study performing docking calculations to evaluate the inhibitory effect of Chinese berry extracts rich in proanthocyanidins on α-amylase found that the extracts induced conformational changes and modified the microenvironment polarity of some residues on the enzyme’s active site, which explain the inhibitory effects of these extracts [12]. The docking calculations found that trimers presented the most efficient binding to the active site of α-amylase [12]. These authors argued that this interaction is more potent than monomers and dimers, as trimers possess more H-bonds, present a stronger hydrophobic effect, and possess more π–π bonds. Nevertheless, oligomers with higher DP lead to superficial interactions that weaken their binding to α-amylase [12].

#### 3.2.2. Inhibition of α-Glucosidase by *Carménère* Pomace Polyphenol Extracts

**Figure 4:**
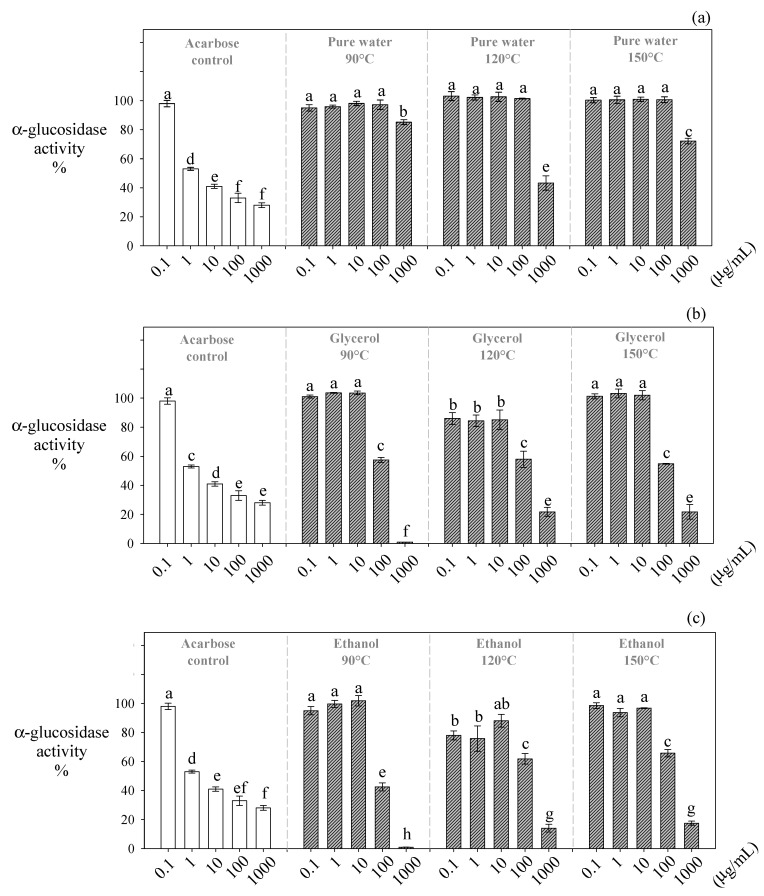
Inhibitory effect of *Carménère* pomace extracts obtained by HPLE at different temperatures using protic solvents on the α-glucosidase activity. (**a**): pure water, (**b**): glycerol, and (**c**): ethanol. The different low-case letters in the bars show the significant inhibitory differences (*p* < 0.05) between the different HPLE temperatures and extract concentrations for the same HPLE solvent.

Water-glycerol and water-ethanol extracts (1000 µg/mL) obtained at 90 °C significantly (*p* ≤ 0.05) decreased the α-glucosidase activity down to ~0.87% and ~1.02%, respectively (Figure 4). These extracts were substantially more effective in inhibiting α-glucosidase than acarbose (~27%) at the same concentration (Figure 3a). Contrarily, the extracts obtained at temperatures higher than 90 °C presented the lowest inhibitory effect (Figure 3b,c).

As discussed above, our ethanolic extracts obtained at 90 °C contained the highest amounts of trimers and tetramers. Like α-amylase, procyanidins with a DP higher than 2 are better inhibiting agents of α-glucosidase than their monomers [40,41]. Guyot et al. [42] and Yilmazer-Musa et al. [11] found that only galloylated procyanidins like B1, B2, and C1 present the ability to inhibit this enzyme. They indicated that the polyphenols´ galloylated groups interact with α-glucosidase, favoring its precipitation and consequently its inhibition. Wang et al. [43] found, in a recent study including docking calculations, that the proanthocyanidins from Chinese bayberry present a remarkable α-glucosidase inhibitory activity through a non-competitive allosteric mechanism. It was found that proanthocyanidins interact with some amino acid residues around the allosteric site of the enzyme, mainly through hydrogen bonds and hydrophobic interactions, which lead to the inhibition of α-glucosidase [43].

Notably, our ethanolic extracts obtained at 90 °C inhibit α-amylase moderately and α-glucosidase strongly. This is an advantage, because in the development of antidiabetic drugs, a higher incidence of gastrointestinal side-effects has been associated with an excessive inhibition of both enzymes [9,10,11,12,13]. The strong inhibition of α-amylase and α-glucosidase leads to high amounts of undigested polymeric sugars reaching the colon and generating gaseous metabolites by bacterial fermentation, which leads to flatulence, abdominal distension, borborygmus, and diarrhea [14].

## 4. Conclusions

The total polyphenol content and antioxidant capacity of our HPLE grape extracts were maximized at high temperatures for the three protic solvents tested. The water-glycerol extracts presented the highest total polyphenol content, antioxidant capacity, and concentration of monomers and dimers at all temperatures. In turn, the water-ethanol extracts presented the highest levels of trimers and tetramers. The *Carménère* pomace extracts (1000 µg/mL) obtained at 90 °C using water-ethanol mixtures inhibited the T2DM-related enzymes significantly. These extracts presented a differential inhibitory effect, which was mild for α-amylase and strong for α-glucosidase. Thus, they can be considered a suitable inhibitor of T2DM-related enzymes because their dual inhibition will probably avoid the undesirable collateral effect of synthetic drugs.

HPLE at moderate temperatures (90 °C) using water-ethanol mixtures is an excellent option to sustainably obtain botanical extracts rich in procyanidins with a selective inhibitory activity against α-amylase and α-glucosidase. Under these conditions, the extraction of procyanidins with a DP higher than 2 is favored while preventing their oxidation and hydrolysis.

Future research in this area should focus on the rational design of an industrial-scale process and the verification of the HPLE extracts’ bioactivity in clinical trials.

## Figures and Tables

**Table 1:** Total polyphenol content of the extracts obtained by HPLE.

Description.	Temperature	Solvent					
		Ethanol (15%)		Glycerol (15%)		Pure Water	
		Mean	CV	Mean	CV	Mean	CV
TPC (mg GAE/g dw)	90 °C	10.05 ^aA^	0.02	11.40 ^bA^	0.02	3.88 ^cA^	0.01
	120 °C	17.02 ^aB^	0.01	18.16 ^aB^	0.01	5.17 ^bB^	0.02
	150 °C	18.40 ^aB^	0.01	20.21 ^bB^	0.01	9.44 ^cC^	0.01

The results are expressed as µg per gram dry weight. CV: coefficient variation. The different capital letters in the same column show significant differences (*p* < 0.05) at different temperatures for the same solvent, while the different low-case letters in the same row show significant differences between the different solvents at the same temperature (*p* < 0.05).

**Table 2:** Antioxidant capacity of the extracts obtained by HPLE.

Description.	Temperature	Solvent					
		Ethanol (15%)		Glycerol (15%)		Pure Water	
		Mean	CV	Mean	CV	Mean	CV
ORAC (µmol TE/g dw)	90 °C	233.59 ^aA^	0.03	248.48 ^aA^	0.05	106.03 ^bA^	0.05
	120 °C	242.87 ^aA^	0.05	288.48 ^bB^	0.04	152.43 ^cB^	0.04
	150 °C	293.81 ^bB^	0.04	314.05 ^aC^	0.05	226.85 ^cC^	0.03
DPPH IC_50_ (mg/mL)	90 °C	69.20 ^aA^	0.02	66.20 ^aA^	0.05	160.53 ^bA^	0.04
	120 °C	38.54 ^aB^	0.04	32.17 ^bB^	0.03	117.25 ^cB^	0.05
	150 °C	23.78 ^aC^	0.03	21.59 ^bC^	0.03	66.20 ^cC^	0.03

The results are expressed as µg per gram dry weight. CV: coefficient variation. The different capital letters in the same column show significant differences (*p* < 0.05) at different temperatures for the same solvent, while the different low-case letters in the same row show significant differences between the different solvents at the same temperature (*p* < 0.05).

**Table 3:** Profile of the specific polyphenols obtained by HPLE.

Polyphenols.	Temperature	Solvent					
		Ethanol (15%)		Glycerol (15%)		Pure Water	
		Mean	CV	Mean	CV	Mean	CV
Monomers (µg/g dw)							
Gallic acid	90 °C	26.40 ^aA^	0.08	29.15 ^aA^	0.12	1.91 ^bA^	0.03
	120 °C	58.32 ^aB^	0.09	57.66 ^aB^	0.11	2.20 ^bA^	0.11
	150 °C	91.47 ^aC^	0.10	99.26 ^aC^	0.08	16.32 ^bB^	0.12
Catechin	90 °C	33.17 ^bA^	0.04	52.77 ^aA^	0.04	26.16 ^bA^	0.03
	120 °C	108.80 ^aB^	0.10	117.05 ^aB^	0.04	36.95 ^bB^	0.14
	150 °C	179.55 ^aC^	0.05	203.60 ^aC^	0.06	126.93 ^bC^	0.12
Epicatechin gallate	90 °C	9.98 ^aA^	0.09	12.32 ^aA^	0.13	3.20 ^bA^	0.12
	120 °C	14.84 ^bB^	0.04	20.52 ^aB^	0.11	3.17 ^cA^	0.07
	150 °C	31.66 ^bC^	0.04	54.61 ^aC^	0.06	17.49 ^cB^	0.10
Quercetin	90 °C	30.08 ^bA^	0.07	39.84 ^aA^	0.12	6.07 ^cA^	0.07
	120 °C	32.50 ^bA^	0.03	55.46 ^aB^	0.04	12.09 ^cB^	0.07
	150 °C	41.45 ^bB^	0.05	92.11 ^aC^	0.07	26.92 ^cC^	0.11
Epicatechin	90 °C	25.26 ^bA^	0.06	65.76 ^aA^	0.04	27.48 ^bA^	0.08
	120 °C	140.77^a B^	0.09	153.05 ^aB^	0.05	33.59 ^bA^	0.12
	150 °C	189.44 ^aC^	0.14	162.45 ^aB^	0.03	81.69 ^bB^	0.15
Kaempferol	90 °C	1.68 ^bA^	0.06	6.48 ^aA^	0.12	0.47 ^cA^	0.03
	120 °C	5.63 ^bB^	0.10	8.72 ^aA^	0.07	0.66 ^cB^	0.03
	150 °C	15.29 ^bC^	0.08	20.02 ^aB^	0.04	1.18 ^cC^	0.04
Rutin	90 °C	1.10 ^aA^	0.06	1.75 ^aA^	0.04	0.42 ^cA^	0.04
	120 °C	1.99 ^aB^	0.07	2.37 ^aB^	0.10	0.65 ^bB^	0.05
	150 °C	ND		ND		ND	
Dimers (µg/g dw)							
Procyanidin B1	90 °C	36.55 ^bB^	0.03	43.08 ^aB^	0.08	14.92 ^cB^	0.11
	120 °C	52.11 ^aC^	0.14	65.99 ^aC^	0.13	12.75 ^bB^	0.08
	150 °C	10.03 ^bA^	0.07	35.08 ^aA^	0.11	4.05 ^cA^	0.11
Procyanidin B2	90 °C	57.63 ^bB^	0.03	72.05 ^aB^	0.07	37.29 ^cB^	0.03
	120 °C	58.06 ^bB^	0.04	87.94 ^aC^	0.04	14.26 ^cA^	0.04
	150 °C	42.56 ^bA^	0.11	61.08 ^aA^	0.10	13.04 ^cA^	0.06
Trimers (µg/g dw)							
Procyanidin C1	90 °C	17.32 ^aB^	0.07	13.46 ^aB^	0.09	2.13 ^bB^	0.03
	120 °C	12.56 ^aA^	0.07	6.49 ^bA^	0.11	1.47 ^cA^	0.09
	150 °C	6.95 ^aC^	0.11	5.95 ^aA^	0.06	ND	
Procyanidin B2 gallate	90 °C	55.81 ^aA^	0.08	48.78 ^aB^	0.06	14.95 ^bC^	0.12
	120 °C	49.17 ^aA^	0.14	42.21 ^aB^	0.12	6.47 ^bB^	0.08
	150 °C	25.32 ^aB^	0.06	16.24 ^bA^	0.08	4.13^cA^	0.08
Tetramers (µg/g dw)							
Procyanidin B2 di-O-gallate	90 °C	17.13 ^aC^	0.07	14.97 ^bC^	0.06	4.35 ^cB^	0.07
	120 °C	12.65 ^aB^	0.09	7.56 ^bB^	0.07	1.89 ^cA^	0.11
	150 °C	7.21 ^aA^	0.04	3.64 ^bA^	0.07	ND	

The results are expressed as µg of specific polyphenols per gram dry weight. CV: variation coefficient. The different capital letters in the same column show the significant differences (*p* < 0.05) between the different HPLE temperatures for the same HPLE solvent, while the different low-case letters in the same row show the significant differences (*p* < 0.05) between the different extraction solvents at the same HPLE temperature.

## Data Availability

Not applicable.

## References

[1] Minio A., Massonnet M., Figueroa-Balderas R., Castro A., Cantu D. (2019). Diploid genome assembly of the wine grape carménère. G3 Genes Genomes Genet..

[2] Fernández K., Kennedy J.A., Agosin E. (2007). Characterization of Vitis vinifera L. Cv. Carmenere Grape and Wine Proanthocyanidins. J. Agric. Food Chem..

[3] Servicio Agrícola y Ganadero S.A.G. Informe Ejecutivo Producción de Vinos. http//www.sag.cl/content/informe-ejecutivo-produccion-de-vinos-2018/.

[4] De la Cerda-Carrasco A., López-Solís R., Nuñez-Kalasic H., Peña-Neira Á., Obreque-Slier E. (2015). Phenolic composition and antioxidant capacity of pomaces from four grape varieties (*Vitis vinifera* L.). J. Sci. Food Agric..

[5] Huaman-Castilla N.L., Mariotti-Celis M.S., Perez-Correa J.R. (2017). Polyphenols of Carménère Grapes. Mini. Rev. Org. Chem..

[6] Rauf A., Imran M., Abu-Izneid T., Patel S., Pan X., Naz S., Sanches Silva A., Saeed F., Rasul Suleria H.A. (2019). Proanthocyanidins: A comprehensive review. Biomed. Pharm..

[7] Scalbert A., Williamson G. (2000). Dietary Intake and Bioavailability of Polyphenols. J. Med. Food.

[8] Tungmunnithum D., Thongboonyou A., Pholboon A., Yangsabai A. (2018). Flavonoids and Other Phenolic Compounds from Medicinal Plants for Pharmaceutical and Medical Aspects: An Overview. Medicines.

[9] Kalita D., Holm D.G., Labarbera D.V., Petrash J.M., Jayanty S. (2018). Aldose Reductase By Potato Polyphenolic Compounds. PLoS ONE.

[10] Kumar S., Narwal S., Kumar V., Prakash O. (2011). α-glucosidase inhibitors from plants: A natural approach to treat diabetes. Pharm. Rev..

[11] Yilmazer-Musa M., Griffith A.M., Michels A.J., Schneider E., Frei B. (2012). Grape seed and tea extracts and catechin 3-gallates are potent inhibitors of α-amylase and α-glucosidase activity. J. Agric. Food Chem..

[12] Wang M., Chen J., Ye X., Liu D. (2020). In vitro inhibitory effects of Chinese bayberry (Myrica rubra Sieb. et Zucc.) leaves proanthocyanidins on pancreatic α-amylase and their interaction. Bioorg. Chem..

[13] Ou-Yang C., Chai W., Xu X., Song S., Wei Q., Huang Q., Zou Z. (2020). Inhibitory potential of proanthocyanidins from the fruit pulp of *Clausena lansium* (Lour.) Skeels against α-glucosidase and non-enzymatic glycation: Activity and mechanism. Process Biochem..

[14] Zhang Y., Santosa R.W., Zhang M., Huo J., Huang D. (2020). Characterization and bioactivity of proanthocyanidins during Malay cherry (*Lepisanthes alata*) fruit ripening. Food Biosci..

[15] Ameer K., Shahbaz H.M., Kwon J.H. (2017). Green Extraction Methods for Polyphenols from Plant Matrices and Their Byproducts: A Review. Compr. Rev. Food Sci. Food Saf..

[16] Monrad J.K., Howard L.R., King J.W., Srinivas K., Mauromoustakos A.S. (2010). Subcritical Solvent Extraction of Procyanidins from Dried Red Grape Pomace. J. Agric. Food Chem..

[17] Petersson E.V., Liu J., Sjöberg P.J.R., Danielsson R., Turner C. (2010). Pressurized Hot Water Extraction of anthocyanins from red onion: A study on extraction and degradation rates. Anal. Chim. Acta.

[18] Ko M.J., Cheigh C.I., Cho S.W., Chung M.S. (2011). Subcritical water extraction of flavonol quercetin from onion skin. J. Food Eng..

[19] Vergara-Salinas J.R., Bulnes P., Zúñiga M.C., Pérez-Jiménez J., Torres J.L., Mateos-Martín M.L., Agosin E., Pérez-Correa J.R. (2013). Effect of pressurized hot water extraction on antioxidants from grape pomace before and after enological fermentation. J. Agric. Food Chem..

[20] Plaza M., Abrahamsson V., Turner C. (2013). Extraction and Neoformation of Antioxidant Compounds by Pressurized Hot Water Extraction from Apple Byproducts. J. Agric. Food Chem.

[21] Huaman-Castilla N.L., Mart M., Camilo C., Pedreschi F., Mariotti-celis M., Ricardo P. (2019). The Impact of Temperature and Ethanol Concentration on the Global Recovery of Specific Polyphenols in an Integrated HPLE/RP Process on Carm é n è re Pomace Extracts. Molecules.

[22] Huamán-Castilla N.L., Mariotti-Celis M.S., Martinez-Cifuentes M., Perez-Correa J.R. (2020). Glycerol as Alternative Co-Solvent for Water Extraction of Polyphenols from Carm é n è re Pomace: Hot Pressurized Liquid Extraction and Computational Chemistry Calculations. Biomolecules.

[23] Monrad J.K., Howard L.R., King J.W., Srinivas K., Mauromoustakos A.S. (2010). Subcritical Solvent Extraction of Anthocyanins from Dried Red Grape Pomace. J. Agric. Food Chem..

[24] Singleton V.L., Rossi J.A., Rossi J. (1965). Colorimetry of Total Phenolics with Phosphomolybdic-Phosphotungstic Acid Reagents. Am. J. Enol. Vitic..

[25] Brand-Williams W., Cuvelier M.E., Berset C. (1995). Use of a Free Radical Method to Evaluate Antioxidant Activity. Food Sci. Technol..

[26] Chirinos R., Campos D., Warnier M., Pedreschi R., Rees J.F., Larondelle Y. (2008). Antioxidant properties of mashua (*Tropaeolum tuberosum*) phenolic extracts against oxidative damage using biological in vitro assays. Food Chem..

[27] Campos D., Teran-Hilares F., Chirinos R., Aguilar-Galvez A., García-Ríos D., Pacheco-Avalos A., Pedreschi R. (2020). Bioactive compounds and antioxidant activity from harvest to edible ripeness of avocado cv. Hass (*Persea americana*) throughout the harvest seasons. Int. J. Food Sci. Technol..

[28] Pacheco L.V., Parada J., Pérez-Correa J.R., Mariotti-Celis M.S., Erpel F., Zambrano A., Palacios M. (2020). Bioactive Polyphenols from Southern Chile Seaweed as Inhibitors of Enzymes for Starch Digestion. Mar. Drugs.

[29] Jessop P.G., Jessop D.A., Fu D., Phan L. (2012). Solvatochromic parameters for solvents of interest in green chemistry. Green Chem..

[30] Mariotti-Celis M.S., Martínez-Cifuentes M., Huamán-Castilla N., Pedreschi F., Iglesias-Rebolledo N., Pérez-Correa J.R. (2018). Impact of an integrated process of hot pressurised liquid extraction–macroporous resin purification over the polyphenols, hydroxymethylfurfural and reducing sugars content of Vitis vinifera ‘Carménère’ pomace extracts. Int. J. Food Sci. Technol..

[31] Thaipong K., Boonprakob U., Crosby K., Cisneros-Zevallos L., Hawkins Byrne D. (2006). Comparison of ABTS, DPPH, FRAP, and ORAC assays for estimating antioxidant activity from guava fruit extracts. J. Food Compos. Anal..

[32] Mariotti-Celis M.S., Martínez-Cifuentes M., Huamán-Castilla N., Vargas-González M., Pedreschi F., Pérez-Correa J.R. (2018). The antioxidant and safety properties of spent coffee ground extracts impacted by the combined hot pressurized liquid extraction–resin purification process. Molecules.

[33] Roy M.K., Koide M., Rao T.P., Okubo T., Ogasawara Y., Juneja L.R. (2010). ORAC and DPPH assay comparison to assess antioxidant capacity of tea infusions: Relationship between total polyphenol and individual catechin content. Int. J. Food Sci. Nutr..

[34] Wijngaard H., Brunton N. (2009). The optimization of extraction of antioxidants from apple pomace by pressurized liquids. J. Agric. Food Chem..

[35] Chaaban H., Ioannou I., Chebil L., Slimane M., Gérardin C., Paris C., Charbonnel C., Chekir L., Ghoul M. (2017). Effect of heat processing on thermal stability and antioxidant activity of six flavonoids. J. Food Process. Preserv..

[36] Jessop P.G. (2011). Searching for green solvents. Green Chem..

[37] Ćurko N., Tomašević M., Bubalo M.C., Gracin L., Redovniković I.R., Ganić K.K. (2017). Extraction of proanthocyanidins and anthocyanins from grape skin by using ionic liquids. Food Technol. Biotechnol..

[38] Yilmazer-Musa M., Griffith A.M., Michels A.J., Schneider E., Balz F. (2015). Inhibition of α-Amylase and α-Glucosidase Activity by Tea and Grape Seed Extracts and their Constituent Catechins. J. Agric. Food Chem..

[39] Lavelli V., Sri Harsha P.S.C., Ferranti P., Scarafoni A., Iametti S. (2016). Grape skin phenolics as inhibitors of mammalian α-glucosidase and α-amylase—Effect of food matrix and processing on efficacy. Food Funct..

[40] Gonçalves R., Mateus N., de Freitas V. (2011). Inhibition of α-amylase activity by condensed tannins. Food Chem..

[41] Kong F., Qin Y., Su Z., Ning Z., Yu S. (2018). Optimization of Extraction of Hypoglycemic Ingredients from Grape Seeds and Evaluation of α-Glucosidase and α-Amylase Inhibitory Effects In Vitro. J. Food Sci..

[42] Guyot S., Pellerin P., Marc Brillouet L., Cheynier V. (1996). Inhibition of β-glucosidase (amygdalae dulces) by (+)-catechin oxidation products and procyanidin dimers. Biosci. Biotechnol. Biochem..

[43] Wang M., Jiang J., Tian J., Chen S., Ye X., Hu Y., Chen J. (2019). Inhibitory mechanism of novel allosteric inhibitor, Chinese bayberry (Myrica rubra Sieb. et Zucc.) leaves proanthocyanidins against α-glucosidase. J. Funct. Foods.

